# Nomograms containing body dose parameters for predicting survival in patients with nasopharyngeal carcinoma

**DOI:** 10.1007/s00405-023-08173-9

**Published:** 2023-08-08

**Authors:** Jianyun Jiang, Ruiping Zhai, Fangfang Kong, Chengrun Du, Hongmei Ying

**Affiliations:** 1 Department of Radiation Oncology, Fudan University Shanghai Cancer Center, 270 Dong An Road, Shanghai, 200032 China; 2 Department of Oncology, Shanghai Medical College, Fudan University, Shanghai, 200032 China; 3 Shanghai Clinical Research Center for Radiation Oncology, Shanghai, China; 4 Shanghai Key Laboratory of Radiation Oncology, Shanghai, 200032 China

**Keywords:** Nasopharyngeal carcinoma, Progression-free survival, Overall survival, Body dose, Intensity-modulated radiation therapy, Dose–volume histogram

## Abstract

**Purpose:**

To assess the impact of body dose on survival outcomes in nasopharyngeal carcinoma (NPC) patients and to create novel nomograms incorporating body dose parameters for predicting survival.

**Methods:**

594 of non-metastasis NPC patients (training group, 396; validation group, 198) received intensity-modulated radiation therapy at our institution from January 2012 to December 2016. Patient characteristics, body dose parameters in dose–volume histogram (DVH) and hematology profiles were collected for predicting overall survival (OS) and progression-free survival (PFS). Nomograms for OS and PFS were developed using the selected predictors. Each nomogram was evaluated based on its C-index and calibration curve.

**Results:**

Body dose-based risk score for OS (RS_OS_), N stage, age, and induction chemotherapy were independent predictors for OS, with a C-index of 0.784 (95% CI 0.749–0.819) in the training group and 0.763 (95% CI 0.715–0.810) in the validation group for the nomogram. As for PFS, the most important predictors were the body dose-based risk score for PFS (RS_PFS_)_,_ N stage, and induction chemotherapy. C-index of PFS nomogram was 0.706 (95% CI 0.681–0.720) in the training group and 0.691 (95% CI 0.662–0.711) in the validation group. The two models outperformed the TNM staging system in predicting outcomes.

**Conclusions:**

Body dose coverage is a useful predictor of prognosis in clinical routine patients. The novel nomograms integrating body dose parameters can precisely predict OS and PFS in NPC patients.

**Supplementary Information:**

The online version contains supplementary material available at 10.1007/s00405-023-08173-9.

## Introduction

Non-metastasis nasopharyngeal carcinoma (NPC) is typically treated with intensity-modulated radiation therapy (IMRT), which delivers a precise dose to the tumor while sparing nearby organs at risk (OARs) [1, 2]. According to NCCN guidelines, locally advanced NPC (LANPC) should be treated with induction chemotherapy (IC) and concurrent chemotherapy (CC), or CC combined with adjuvant chemotherapy (AC) [3]. Despite advances in radiotherapy, tumor relapse still occurs in 20–30%, and the 5-year estimated overall survival rate for NPC is 84.0% [4, 5]. Moreover, salvage therapy has a poor survival rate for patients who relapse after definitive radiotherapy [6–8]. A comprehensive and accurate risk staging model is urgently needed to improve patient prognosis and guide treatment decisions.

Radiation dosage is a crucial clinical indicator for tumor control and complication assessment. Dose planning parameters can help predict radiotherapy benefits and risks for patients. IMRT feasibility has been evaluated using some metrics [9]. Previous studies have examined the impact of dosimetric parameters on NPC prognosis [10], but did not consider all potentially valuable indicators in the dose–volume histograms (DVHs). Moreover, most studies focused solely on relationships between local recurrence (LR) and dose of the planned target volumes (PTVs) [11–14]. There is limited research on the association between dosimetric factors and long-term overall survival (OS). Furthermore, volume-based DVH metrics are ideal for assessing radiation-induced effects on hematology profiles of patients [15]. Hence, we used the least absolute shrinkage and selection operator (LASSO) [16, 17] to evaluate meaningful DVH parameters and obtained body dose-based risk scores for OS (RS_OS_) and PFS (RS_PFS_). Our goal was to develop nomograms that integrate clinical characteristics and therapeutic DVH parameters to determine the probabilities of OS and PFS at 3 and 5 years for IMRT-treated NPC patients.

## Materials and methods

### Patients and treatments

Clinical data from treatment-naive NPC patients at Fudan University Shanghai Cancer  Center between January 2012 and December 2016 were analyzed. This study was approved by the Institutional Review Board of Fudan University Shanghai Cancer Center (No. 1612167-18). All participants gave informed consent prior to participation.

All patients received definitive IMRT. Inclusion criteria were: pathologically confirmed NPC (according to AJCC/UICC staging system, 7th edition); absence of metastasis at diagnosis; follow-up period longer than 6 months; and complete RT course without delay. Any patients without radiotherapy or complete clinical and radiotherapy planning data were excluded from the study. 594 patients were randomly allocated to a training group and a validation group in a 2:1 ratio using table of randomized number, with patient characteristics including age, gender, TNM stage, T classification, N classification, chemotherapy regimens, and prescription dose to the PTV extracted. Absolute values of blood indicators, including pretreatment white blood cell (pre-WBC), lymphocyte (pre-ALC), neutrophil (pre-ANC), monocyte (pre-AMC), platelet (pre-PLT), lactate dehydrogenase (pre-LDH) levels, and albumin (pre-ALB) levels were assessed within 7 days before radiotherapy.

The majority of stage I–II patients (T1N0, T1N1 and T2N0) received IMRT once per day, five times per week. Patients with T2–4 and N + received IMRT plus cisplatin-based chemotherapy (IC, CC and AC) intravenously every 3 weeks using TPF, GP, and TP regimens.

### Target volume delineation and dose prescription

Optimization and evaluation of the treatment plan were carried out with Philips Pinnacle treatment planning system (TPS; version 8.0; Fitchburg, WI, USA). Patients underwent CT simulation with contrast-enhancement, wearing thermoplastic masks for the head, neck, and shoulders. All patients were scanned with serial slices of 5 mm from the vertex to the clavicle (Supplementary Fig. 1). The target volumes were delineated on the CT scans, which were then reevaluated by the same clinician and the same radiologist. The target volumes were defined according to International Commission on Radiation Units and Measurements Report 50 and Report 62. Primary nasopharyngeal tumors (GTV-P) and lymph nodes (GTV-LN) were included in the gross tumor volume (GTV).

Clinical high-risk nasopharyngeal tumor volume (CTV1) was subclinical disease, including GTV with margins of not less than 8 mm, such as nasopharyngeal cavity, posterior third of nasal cavity and maxillary sinus, parapharyngeal space, pterygopalatine fossa, lateral pterygial plate, skull base, prevertebral muscle, anterior half or two-thirds of the slope (all in cases of invasion), and at least half of the sphenoid sinus (all for T3 or T4 lesions). High-risk lymphatic drainage areas include bilateral retropharyngeal lymph nodes, bilateral upper neck lymph nodes (grade II, III, VA), and positive lymph nodes in the ipsilateral lower neck lymphatic areas. The low-risk lymphatic drainage area (CTV2) includes the lymphatic area within the lower neck with no positive lymph nodes (generally grade IV and VB). For N0 patients, neither grade IV nor VB radiation was exposed. On the basis of GTV-P, GTV-LN, CTV1 and CTV2, add 3–5 mm allowance, respectively, to create the planned target volumes (PTV-G, PTV-LN, PTV1 and PTV2).

For T1–2 disease, the prescribed doses of PTV-G and PTV-LN were 66 Gy (30 fractions) and the prescribed doses of PTV1 and PTV2 were 60 Gy and 54 Gy (30 fractions). For T3–4 disease, a total of 70.4 Gy was delivered in 32 fractions to PTV-G, 66 Gy in 32 fractions to PTV-LN, 60 Gy and 54 Gy in 32 fractions to PTV1 and PTV2. PTV volume less than 95% of the prescription dose should not exceed 1%. More than 110% of the prescription dose was not allowed inside or outside the PTV. Simultaneous integrated boost technique was applied to all target volumes. The doses to these OARs were limited as much as possible without sacrificing PTV coverage. The ideal maximal point dose should not exceed 54 Gy for brainstem, optic chiasma and optic nerve, 45 Gy for spinal cord, and 60 Gy for temporal lobe. However, if these limits cannot be met, acceptable criteria were less than 60 Gy to 1% volume for brainstem, optic chiasma and optic nerve, and less than 50 Gy to 1 cc for spinal cord, and less than 65 Gy maximal point dose for temporal lobe.

### Dosimetric data extraction

We extracted DVH parameters for each patient, including mean body dose (MBD), integral body dose (IBD), and Vd from V5 to V70 in 5 steps. Vd (%) indicated the proportion of the body receiving at least d Gy. MBD represented the average radiation dose absorbed by the body as examined by CT images during the IMRT course. IBD was the product of MBD and the overall volume of the CT scan. LASSO, a regression method that adjusts *λ* to remove important parameters and high collinearity among DVH metrics [16], ultimately screens for features related to survival conditions. Body dose-based risk scores for OS (RS_OS_) and PFS (RS_PFS_) were calculated for each patient by summing the selected features weighted by their respective coefficients in LASSO.

### Nomogram development and validation

We used LASSO-selected features and clinical data to perform univariate Cox regression analysis in the training group, evaluating the predictive ability of RS_OS_ and RS_PFS_. Factors with a *P* value < 0.1 were included in multivariate analysis using backward likelihood method to identify key indicators (*P* < 0.05). Finally, we established OS and PFS nomograms based on a multivariate Cox analysis using “rms” R package.

To assess the discrimination ability of the nomogram, we used Harrell’s concordance index (C-index) with 1000 bootstrap resamples and performed time-dependent receiver operating characteristic curve (tdROC) analysis. We compared the area under ROC curves (AUC) between nomograms and TNM stage for predicting OS and PFS using the “survivalROC” package in R software. Calibration curves were used to compare observed OS and PFS with predicted probability from the nomogram."

### Classification of patients based on risk

The R package “maxstat” (version 0.7–25, https://CRAN.R-project.org/package=maxstat) was used to determine the optimal cut point for the risk score of each nomogram in the training cohort. Patients were then classified into high-risk and low-risk groups based on this threshold, and their survival was compared using the “survfit” function of R in the two risk groups.

### Outcomes and follow-up

The median duration of follow-up time was 82 months (range 8.9–126.4 months). Progression-free survival (PFS) is the time from treatment initiation until disease progression or death. The overall survival (OS) is the time from treatment initiation to death or last follow-up.

### Statistical analysis

We used IBM SPSS and R software for statistical analysis. LASSO was applied to screen important parameters related to PFS or OS using the “glmnet” package in R. A tenfold cross-validation was performed, and significant parameters were identified by selecting the *λ* with the smallest deviance. Nomograms were generated using the “rms” package, while Kaplan–Meier survival curves were plotted using the “survival” and “survminer” packages.

## Results

### Patient characteristics

The patient characteristics of both cohorts were presented in Table 1. Among the 594 patients, the median age was 48 years (range 14–77 years), with males accounting for 73.9% and females accounting for 26.1%. Stage I, stage II, stage III and stage IVA patients accounted for 0.7%, 13.5%, 33.8% and 52.0%, respectively. The median counts of pretreatment white blood cell, lymphocyte, monocyte, neutrophil, platelet, lactate dehydrogenase and albumin were 5.60 × 10^9^/L, 1.70 × 10^9^/L, 0.50 × 10^9^/L, 3.30 × 10^9^/L, 198.00 × 10^9^/L, 201.00U/L and 43.90g/L, respectively. The estimated PFS rates were 79.8% (3 years) and 69.2% (5 years); OS rates were 84.5% (3 years) and 70.7% (5 years).

**Table 1:** Baseline characteristics of the patients

Characteristic	Overall (*n* = 594)	Training cohort (*n* = 396)	Validation cohort (*n* = 198)	*P* value
Gender, *n* (%)				0.472
Female	155 (26.1%)	293 (74.0%)	146 (73.7%)	
Male	439 (73.9%)	103 (26.0%)	52 (26.3%)	
Age, mean (SD)	48.10 (12.33)	48 (12.28)	48.5 (12.45)	0.754
TNM stage, *n* (%)				0.330
I	4 (0.7%)	2 (0.5%)	2 (1.0%)	
II	80 (13.5%)	50 (12.6%)	30 (15.2%)	
III	201(33.8%)	143 (36.1%)	58 (29.3%)	
IV	309 (52.0%)	201 (50.8%)	108 (54.5%)	
T classification, *n* (%)				0.853
T1	46 (7.7%)	29 (7.3%)	17 (8.6%)	
T2	201 (33.8%)	132 (33.3%)	69 (34.8%)	
T3	206 (34.7%)	141 (35.6%)	65 (32.8%)	
T4	141 (23.8%)	94 (23.7%)	47 (23.7%)	
N classification, *n* (%)				0.193
N0	22 (3.7%)	16 (4.0%)	6 (3.0%)	
N1	160 (26.9%)	111 (28.0%)	49 (24.7%)	
N2	209 (35.2%)	145 (36.6%)	64 (32.3%)	
N3	203 (34.2%)	124 (31.3%)	79 (39.9%)	
IC, *n* (%)				0.363
No	237 (39.9%)	152 (38.4%)	85 (42.9%)	
Yes	357 (60.1%)	244 (61.6%)	113 (57.1%)	
CC, *n* (%)				< 0.001*
No	460 (77.4%)	289 (73.0%)	171 (86.4%)	
Yes	134 (22.6%)	107 (27.0%)	27 (13.6%)	
AC, *n* (%)				0.011*
No	369 (62.1%)	261 (65.9%)	108 (54.5%)	
Yes	225 (37.9%)	135 (34.1%)	90 (45.5%)	
Prescription dose, *n* (%)				0.857
66	142 (23.9%)	96 (24.2%)	46 (23.2%)	
70.4	452 (76.1%)	300 (75.8%)	152 (76.8%)	
Pre-WBC, median (IQR)	5.6 (4.3, 7)	5.6 (4.5, 7)	5.5 (4.4, 6.8)	0.399
Pre-ALC, median (IQR)	1.7 (1.3, 2)	1.7 (1.3, 2.1)	1.7 (1.3, 2)	0.834
Pre-AMC, median (IQR)	0.5 (04, 0.6)	0.4 (0.3, 0.6)	0.5 (0.4, 0.6)	0.009*
Pre-ANC, median (IQR)	3.3 (2.6, 4.5)	3.4 (2.5, 4.5)	3.25 (2.27, 4.4)	0.266
Pre-PLT, median (IQR)	198 (158.75, 255)	193 (157.75, 243)	206 (161.75, 271)	0.088
Pre-LDH, median (IQR)	201 (162.25, 420.5)	197.5 (159.25, 410.75)	217 (168.25, 441)	0.050
Pre-ALB, median (IQR)	43.90 (35.4, 50.4)	43.85 (35.4, 50.6)	43.95 (35.8, 50.2)	0.812

*IC* induction chemotherapy, *CC* concurrent chemotherapy, *AC* adjuvant chemotherapy, *Pre-WBC* pretreatment white blood cell count, *Pre-ALC* pretreatment lymphocyte count, *Pre-AMC* pretreatment monocyte count, *Pre-ANC* pretreatment neutrophil count, *Pre-PLT* pretreatment platelet count, *Pre-LDH* pretreatment lactate dehydrogenase level, *Pre-ALB* pretreatment albumin level

*P value $$<$$ 0.05

### Signature construction

**Fig. 1:**
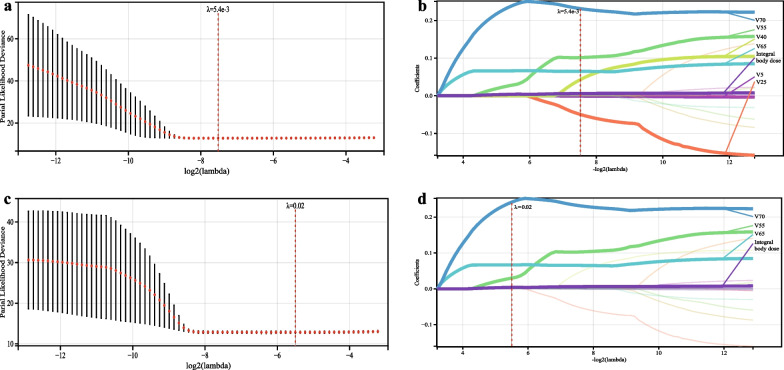
Feature selection using LASSO with *λ* adjusted by the lowest partial-likelihood deviance. Selected significant Vd% parameters were incorporated for overall survival (**a**, **b**) and progression-free survival (**c**, **d**)

Figure 1 displays the hyperparameter *λ* results after a tenfold cross-validation. Among the 16 metrics, 7 metrics were correlated with OS in the training cohort and 4 metrics were associated with PFS. The RS_OS_ and RS_PFS_ risk scores were obtained by summing selected metrics multiplied by their respective coefficients (Supplementary Tables 2 and 3).

### Nomogram establishment and validation

**Fig. 2:**
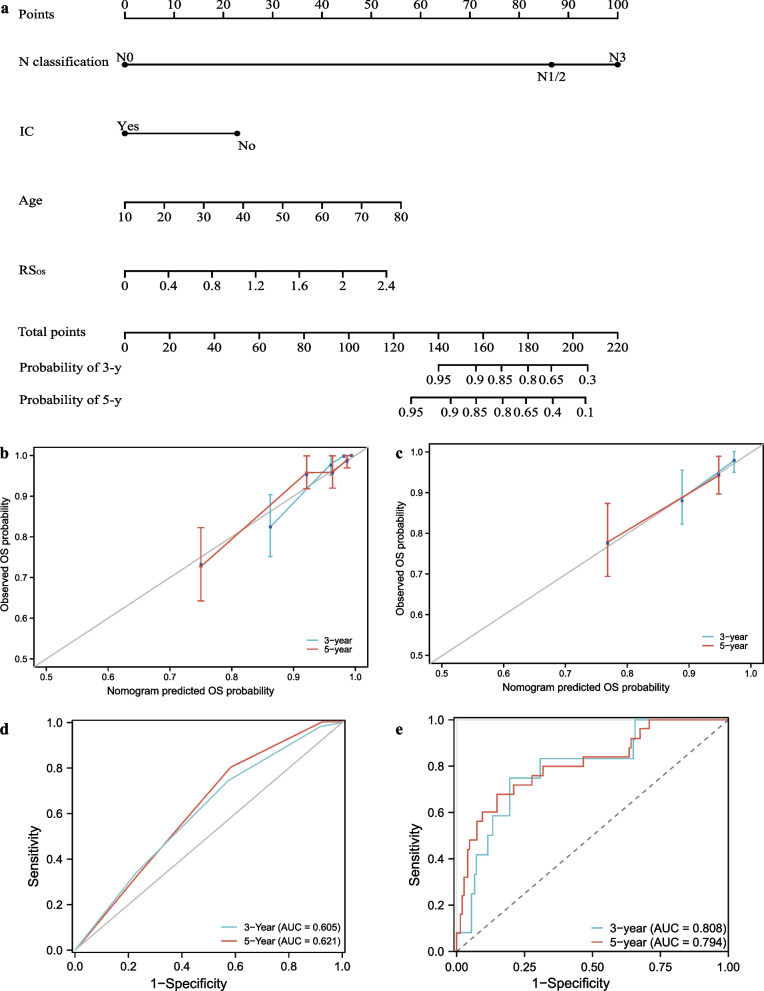
Prediction performance of overall survival (OS) nomogram. The scale on each variable’s line segment shows its possible range of values for obtaining a corresponding score; adding all variable together yields the total score, which predicts the probability of OS survival in 3 or 5 years (**a**). Calibration curves of OS nomogram in the training group (**b**) and validation group (**c**). Area under the receiver operating characteristic curve (AUC) of TNM stage (**d**) and nomogram (**e**) in the validation group. *N* node, *IC* induction chemistry, *RS*_*OS*_ body dose-based risk score for OS

Age, T4 classification (vs T1), N3 classification (vs N0), IC, AC, pre-ALC, pre-ALB and RS_OS_ were significant predictors of OS in the univariate Cox analysis (Table 2) (for all, *P* < 0.1). Multivariate analysis identified age, N classification, IC, and RS_OS_ as independent factors for OS. 3-year and 5-year probabilities of OS were visually quantified based on coefficients of risk factors (Fig. 2a). The calibration curves of OS nomogram demonstrated excellent predictive accuracy (Fig. 2b, c). In the training and validation groups, the nomogram achieved a C-index of 0.784 (95% CI 0.749–0.819) and 0.763 (95% CI 0.715–0.810), respectively, surpassing the TNM staging system’s C-index of 0.613 (95% CI 0.584–0.642) and 0.597 (95% CI 0.546–0.649).

**Table 2:** Univariate analysis and multivariate analysis for predictors of overall survival

Characteristics	Univariate analysis		Multivariate analysis	
	Hazard ratio (95% CI)	P value	Hazard ratio (95% CI)	P value
Gender				
Male	Reference			
Female	0.866 (0.482–1.564)	0.641		
Age	1.055 (1.027–1.084)	< 0.001*	1.046 (1.020–1.073)	< 0.001*
T classification				
T1	Reference		Reference	
T2	2.756 (0.355–3.407)	0.332	2.393 (0.307–3.665)	0.405
T3	4.209 (0.560–6.629)	0.163	3.779 (0.492–5.016)	0.201
T4	6.075 (0.807–8.754)	0.080*	5.095 (0.650–7.914)	0.121
N classification				
N0	Reference			
N1	1.812 (0.890–3.690)	0.211	1.751 (1.026–3.032)	0.048*
N2	1.969 (0.999–3.876)	0.207	1.815 (1.053–3.710)	0.034*
N3	2.136 (1.069–4.267)	0.032*	2.427 (1.155–5.090)	< 0.001*
IC				
No	Reference		Reference	
Yes	0.288 (0.158–0.528)	< 0.001*	0.210 (0.111–0.397)	< 0.001*
CC				
No	Reference			
Yes	0.749 (0.377–1.487)	0.409		
AC				
No	Reference			
Yes	0.535 (0.265–1.079)	0.081*	0.668 (0.316–1.411)	0.290
RS_OS_	3.144 (1.543–6.407)	0.002*	2.821 (1.144–6.956)	0.024*
Pre-WBC	1.012 (0.895–1.145)	0.847		
Pre-ALC	0.635 (0.370–1.089)	0.099*	0.811 (0.475–1.385)	0.443
Pre-AMC	1.746 (0.596–5.114)	0.310		
Pre-ANC	1.023 (0.898–1.166)	0.728		
Pre-PLT	0.999 (0.995–1.003)	0.587		
Pre-LDH	0.999 (0.997–1.002)	0.591		
Pre-ALB	0.926 (0.852–1.007)	0.073*	0.960 (0.869–1.061)	0.422

*CI* confidence intervals, *HR* hazard ratio, *IC* induction chemotherapy, *CC* concurrent chemotherapy, *AC* adjuvant chemotherapy, *RS*_*OS*_ body dose-based risk score for OS, *Pre-WBC* pretreatment white blood cell count, *Pre-ALC* pretreatment lymphocyte count, *Pre-AMC* pretreatment monocyte count, *Pre-ANC* pretreatment neutrophil count, *Pre-PLT* pretreatment platelet count, *Pre-LDH* pretreatment lactate dehydrogenase level, *Pre-ALB* pretreatment albumin level

*P value $<$ 0.1 in the univariate analysis and $<$ 0.05 in the multivariate analysis

Figure 2d, e shows the ROC curves of the TNM staging system and the predictive model. In the training cohort, the OS nomogram (3-year OS: AUC, 0.904, 95% CI 0.851–0.958; 5-year OS: AUC, 0.835, 95% CI 0.771–0.909) was better than TNM staging system (3-year OS: AUC, 0.625, 95% CI 0.584–0.711; 5-year OS: AUC, 0.630, 95% CI 0.576–0.713). In the validation cohort; the 3-year and 5-year AUC of OS nomogram were 0.808 (95% CI 0.761–0.907) and 0.794 (95% CI 0.734–0.892), and that of TNM staging system was 0.605 (95% CI 0.516–0.673) and 0.621 (95% CI 0.537–0.706).

**Fig. 3:**
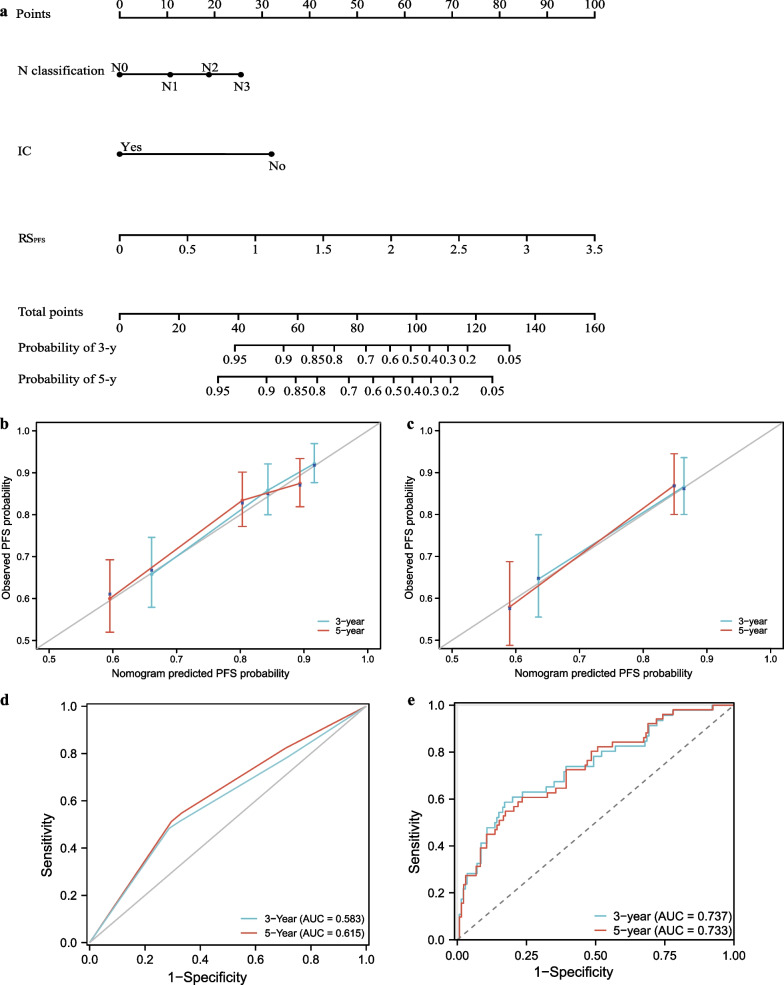
Prediction performance of progression-free survival (PFS) nomogram. The scale on each variable’s line segment shows its possible range of values for obtaining a corresponding score; adding all variable together yields the total score, which predicts the probability of PFS survival in 3 or 5 years (**a**). Calibration curves of PFS nomogram in the training group (**b**) and validation group (**c**). Area under the receiver operating characteristic curve (AUC) of TNM stage (**d**) and nomogram (**e**) in the validation group. *N* node, *IC* induction chemistry, *RS*_*PFS*_ body dose-based risk score for PFS

As for PFS, RS_PFS_, N classification, and IC were the most important predictors (Table 3). The PFS nomogram had a C-index of 0.706 (95% CI 0.681–0.720) in the training group and 0.691 (95% CI 0.662–0.711) in the validation group (Fig. 3a), outperforming TNM staging system’s C-index of 0.639 (95% CI 0.602–0.677) and 0.617 (95% CI 0.579–0.665), respectively; Calibration curves are shown in Fig. 3b, c. The nomogram showed higher AUC value for 3-year PFS (0.745, 95% CI 0.698–0.801) and 5-year PFS (0.733, 95% CI 0.691–0.810) in the training group compared to the TNM staging system (3-year PFS: 0.625, 95% CI 0.531–0.659; 5-year PFS: 0.640, 95% CI 0.554–0.690). Similarly, in the validation group, the nomogram showed better predictive performance than the TNM staging system for both 3-year PFS (0.728 vs 0.583) and 5-year PFS (0.700 vs 0.615) (Fig. 3d, e).

**Table 3:** Univariate analysis and multivariate analysis for predictors of progression-free survival

Characteristics	Univariate analysis		Multivariate analysis	
	Hazard ratio (95% CI)	*P* value	Hazard ratio (95% CI)	*P* value
Gender				
Male	Reference			
Female	0.647 (0.392–1.069)	0.089*	0.650 (0.392–1.077)	0.094
Age	1.008 (0.991–1.026)	0.341		
T classification				
T1	Reference			
T2	1.090 (0.450–2.642)	0.848		
T4	1.695 (0.703–4.084)	0.240		
T3	1.262 (0.530–3.007)	0.599		
N classification				
N0	Reference			
N1	1.783 (1.106–2.776)	0.015*	1.368 (1.183–2.244)	0.013*
N2	2.049 (1.119–2.961)	0.009*	1.942 (1.423–2.523)	0.003*
N3	2.202 (1.290–3.759)	0.003*	2.242 (1.478–3.403)	0.002*
IC				
No	Reference			
Yes	0.615 (0.411–0.920)	0.018*	0.401 (0.261–0.615)	< 0.001*
CC				
No	Reference			
Yes	1.016 (0.650–1.586)	0.946		
AC				
No	Reference			
Yes	0.870 (0.565–1.340)	0.529		
RS_PFS_	3.486 (2.273–5.348)	< 0.001*	3.534 (2.201–5.674)	< 0.001*
Pre-WBC	1.002 (0.920–1.093)	0.956		
Pre-ALC	0.900 (0.603–1.330)	0.352		
Pre-AML	1.670 (0.787–3.540)	0.181		
Pre-ANC	1.010 (0.863–1.074)	0.496		
Pre-PLT	1.001 (0.999–1.004)	0.358		
Pre-LDH	1.001 (0.999–1.002)	0.464		
Pre-ALB	0.968 (0.912–1.026)	0.273		

*CI* confidence intervals, *HR* hazard ratio, *IC* induction chemotherapy, *CC* concurrent chemotherapy, *AC* adjuvant chemotherapy, *RS*_*OS*_ body dose-based risk score for OS, *Pre-WBC* pretreatment white blood cell count, *Pre-ALC* pretreatment lymphocyte count, *Pre-AMC* pretreatment monocyte count, *Pre-ANC* pretreatment neutrophil count, *Pre-PLT* pretreatment platelet count, *Pre-LDH* pretreatment lactate dehydrogenase level, *Pre-ALB* pretreatment albumin level

*P value $<$ 0.1 in the univariate analysis and $<$ 0.05 in the multivariate analysis

Identifying patients at high risk and low risk.

**Fig. 4:**
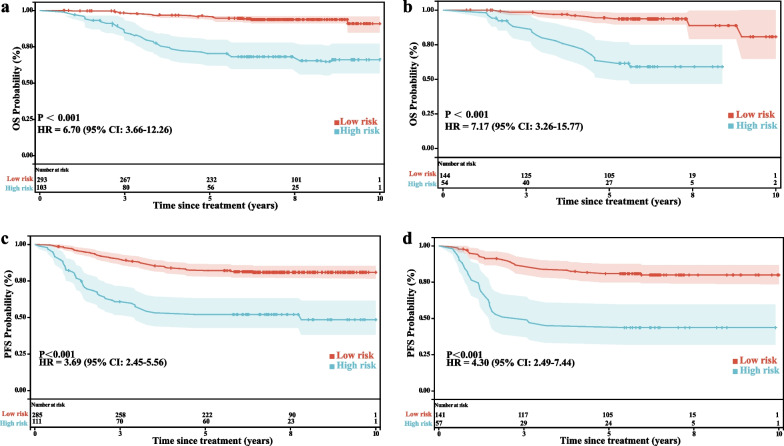
Kaplan–Meier survival curves of training group (**a**, **c**) and validation group (**b**, **d**) to estimate OS (**a**, **b**) and PFS (**c**, **d**) of high-risk and low-risk groups. *CI* confidence intervals, *HR* hazard ratio

The OS and PFS nomograms had optimal cutoff points of 0.845 and 0.336, respectively. Patients with linear prediction scores above the cutoff point were classified as high-risk group. In both the training and validation groups, the K–M survival curves demonstrated that the high-group had significantly worse OS (Fig. 4a, b) and PFS (Fig. 4c, d) than the low-risk group (for both, *P* < 0.001).

## Discussion

To our knowledge, no any previous study has shown a correlation between the body dose parameters and patient outcomes in NPC. We established and validated nomograms to predict OS and PFS in NPC patients, with age, N classification, IC, and RS_OS_ as independent variables for OS prediction; N classification, IC, RS_PFS_ for PFS prediction. Compared to the 7th AJCC TNM staging system, both nomograms significantly outperformed in predicting OS and PFS. Nomogram-derived risk scores allowed us to classify patients into low-risk and high-risk groups.

Relationships between radiation dose and disease prognosis were not examined until Mijnheer et al. [18] and Wittkamper et al.[19]. The ICRU 83 report suggests D50 as a dose–volume parameter for evaluating IMRT plans, but its variability was significant across treatment institutes and techniques [20, 21]. Zhang et al. proposed that D90 is a key DVH indicator for local–regional recurrence of NPC [10], which Xiao et al. identified D95, D5 and homogeneity index as significant dosimetric predictors [22]. However, much of the discussion on NPC prognosis focuses on high-dose PTVs, without considering potential confounding variables, such as immune function, inflammation index, and nutrition status.

The counts of certain blood cells can reflect the balance between the host immune function and tumor function [23]. Pretreatment ALC has been found to be a significant predictor in some studies [24, 25]. Inflammation response and nutritional status have also been explored in relation to NPC outcomes [26–30]. However, these results were inconsistent due to patient population heterogeneity. Therefore, we assessed patients’ hematology profiles by recording immune cell counts and biochemical indicators prior to radiotherapy.

In our study, these pretreatment cell counts did not show statistical significance in multivariate analysis. this may be due to our inclusion of non-metastatic NPC with stages I through IVA, while previous studied focused on specific TNM stages. In addition, we may have overlooked the impact of analyzing these indicators as continuous variables. Many studies have converted cell counts from continuous to categorical variables using historical cutoff points or sample medians, which can result in a false correlation [31]. Pretreatment LDH levels and ALB levels were analyzed in our analysis, as they have been reported to affect NPC survival [32–35]. Although ALB and ALC showed statistical significance in univariate analyses, their significance was not observed in multivariate analyses that included patient demographics, treatment-related parameters, and tumor characteristics.

LASSO analysis identified several dose–volume parameters that impact the survival of NPC patients. The strongest prognosis correlations were found with V70, V65, V55, which carried the greatest weight in the RS equation (Supplementary Tables 2 and 3). These three parameters roughly correspond to the target volumes receiving prescribed doses. As high-dose target volumes increase, OS and PFS decreases progressively. This is justifiable as patients with advanced disease, which may lead to unfavorable prognoses, necessitate larger target volumes and higher prescription doses.

Of note, IBD carries more weight in these two equations (Supplementary Tables 2 and 3). It has been considered to be a more reliable dosimetric parameter that incorporated both low- and high-dose distribution compared to MBD and gross tumor volume [36]. Besides, assuming IBD represents the average dose for large blood vessels may aid in determining the relative radiation exposure of immune cells circulating the irradiated area [37]. Radiation can destroy immune cells, leading to immunosuppressive effects [38], Fractionated long-course radiation therapy, regardless of concurrent chemotherapy type, often results in severe lymphopenia and significantly reduces OS and PFS in various malignancies [39–41]. Based on our findings, higher IBD values were associated with poorer OS and PFS. This suggest that the radiotherapy may compromise the immune system and lead to disease progression by impairing disease surveillance. Further research is needed to confirm this hypothesis.

Furthermore, V5 and V25 act as protection factors in the RS_OS_. IMRT failures are primarily due to distant metastasis, which is responsible for most cancer-related deaths. Increasing the volume of the low-dose region can increase overall absorbed dose and potentially control micro-metastases. Therefore, we suggest expanding irradiated body areas with low doses. IC was found to be a protective factor in both nomograms, as it is believed to aid in the eradication of micro metastases [42].

Compared to TNM staging, which only depicts the anatomical extent of tumors, our nomograms containing body dose parameters and other prognostic-related variables, may also partially estimate potentially immune-toxic radiation doses to the circulating blood pool. This better assist in patients stratifying and optimizing prescription or treatment planning approaches.

Our study has certain limitations. First, the nomograms require external validations as they have only been validated internally. In addition, retrospective studies are susceptible to selection bias due to specific inclusion criteria for patients. Thirdly, most patients’ EBV DNA values were unknown and thus further improvement of the nomogram can be achieved by accounting for this factor. Fourth, we utilized a relatively straightforward metric—radiation dosage to body regions to construct our prognostic nomograms. Further research into more intricate metrics, such as absorbed doses to individual organs, is necessary.

## Conclusion

We have developed and validated novel nomograms containing body dose-based DVH signatures that effectively predicted OS and PFS in non-metastatic NPC patients. Moving forward, greater emphasis should be paced on volume-based metrics in treatment planning to optimize outcomes for NPC patients.

### Supplementary Information

Below is the link to the electronic supplementary material.

Supplementary file1 (DOCX 27 KB)

Supplementary Fig. 1 CT scan images of one of the patients with delineated boundaries in the treatment planning system, including transverse, sagittal, and coronal views)

## Data Availability

The data supporting the findings of this study are not publicly available due to internal institutional policies, but can be obtained from the corresponding author upon reasonable request.
