# Supplementary material for: Nomograms containing body dose parameters for predicting survival in patients with nasopharyngeal carcinoma
Source: Eur Arch Otorhinolaryngol. 2023 Aug 8;281(1):181–92. doi: 10.1007/s00405-023-08173-9 (PMC10764493; doi:10.1007/s00405-023-08173-9)
Supplement: Supplementary file 1 — Supplementary file1 (DOCX 27 KB) [file 405_2023_8173_MOESM1_ESM.docx]

**Nomograms with body dose parameters predict survival in patients with nasopharyngeal carcinoma**

**Journal name:**

Jianyun Jiang, PhD *^1,2,3,4^, Ruiping Zhai, PhD *^1,2,3,4^, Fangfang Kong, PhD ^1,2,3,4^, Chengrun Du, PhD ^1,2,3,4^, Hongmei Ying, PhD ^1,2,3,4^

1.Department of Radiation Oncology, Fudan University Shanghai Cancer Center, Shanghai 200032, China

2.Department of Oncology, Shanghai Medical College, Fudan University, Shanghai 200032, China

3.Shanghai Clinical Research Center for Radiation Oncology

4.Shanghai Key Laboratory of Radiation Oncology, Shanghai 200032, China

*：These authors contributed equally to this work.

**Corresponding author**: Dr. Hongmei Ying

ORCiD: 0000-0003-2642-3135

Telephone number: 13817502024

Postal code: 200032

Mailing address: Fudan University Shanghai Cancer Center, 270 Dong An Road, Shanghai, China.

E-mail address: yinghongmei2020@163.com

**Supplementary Table 1** Mean and standard deviation of body dose parameters

| Body dose parameters | Mean $\pm$ standard deviation |
| --- | --- |
| †mean body dose (cGy) | 2385.9 ± 475.9 |
| †Integral body dose（l.Gy） | 190.4 ± 39.5 |
| †V5 (%) | 72.4 ± 26.6 |
| V10 (%) | 62.1 ± 10.3 |
| V15 (%) | 54.5 ± 9.6 |
| V20 (%) | 47.9 ± 8.6 |
| V25 (%) | 41.9 ± 7.6 |
| V30 (%) | 36.0 ± 6.5 |
| V35 (%) | 30.3 ± 5.5 |
| V40 (%) | 24.8 ± 4.8 |
| V45 (%) | 20.0 ± 3.9 |
| V50 (%) | 16.5 ± 3.3 |
| V55 (%) | 12.7 ± 3.1 |
| V60 (%) | 8.71 ± 2.2 |
| V65 (%) | 3.8 ± 1.9 |
| V70 (%) | 1.4 ± 0.9 |

†Footnotes: V_dGy_ (%) was defined as the minimum absorbed dose that covers x% of the volume of the target; integral body dose was defined as integral dose of the body region receiving radiation.

**Supplementary Table 2** Seven metrics from LASSO analysis were used to construct model RS_OS_.

| Metrics | Coefficient | Score |
| --- | --- | --- |
| †V5 | -0.00103705575646794 | -0.00103705575646794*V5 (%) |
| V25 | -0.0496550437338329 | -0.0496550437338329*V25 (%) |
| V40 | 0.0430972708355125 | 0.0430972708355125*V40 (%) |
| V55 | 0.101451864503953 | 0.101451864503953*V55 (%) |
| V65 | 0.0650045278465668 | 0.0650045278465668*V65 (%) |
| V70 | 0.231516334835631 | 0.231516334835631*V70 (%) |
| Integral body dose | 0.00605232791731264 | 0.00605232791731264*Integral body dose (l.Gy) |

†Footnotes: V_dGy_ (%) was defined as the minimum absorbed dose that covers x% of the volume of the target; integral body dose was defined as integral dose of the body region receiving radiation.

**Supplementary Table 3** Four metrics from LASSO analysis were used to construct model RS_PFS_.

| Metrics | Coefficient | Score |
| --- | --- | --- |
| †V55 | 0.0299726666828279 | 0.0299726666828279*V55 (%) |
| V65 | 0.0666965033349576 | 0.0666965033349576*V65 (%) |
| V70 | 0.241761666768037 | 0.241761666768037*V70 (%) |
| Integral body dose | 0.00408512814863764 | 0.00408512814863764*Integral body dose (l.Gy) |

†Footnotes: V_dGy_ (%) was defined as the minimum absorbed dose that covers x% of the volume of the target; integral body dose was defined as integral dose of the body region receiving radiation.
