# Supplementary figures and images for: Nomograms containing body dose parameters for predicting survival in patients with nasopharyngeal carcinoma
Source: Eur Arch Otorhinolaryngol. 2023 Aug 8;281(1):181–92. doi: 10.1007/s00405-023-08173-9 (PMC10764493; doi:10.1007/s00405-023-08173-9)

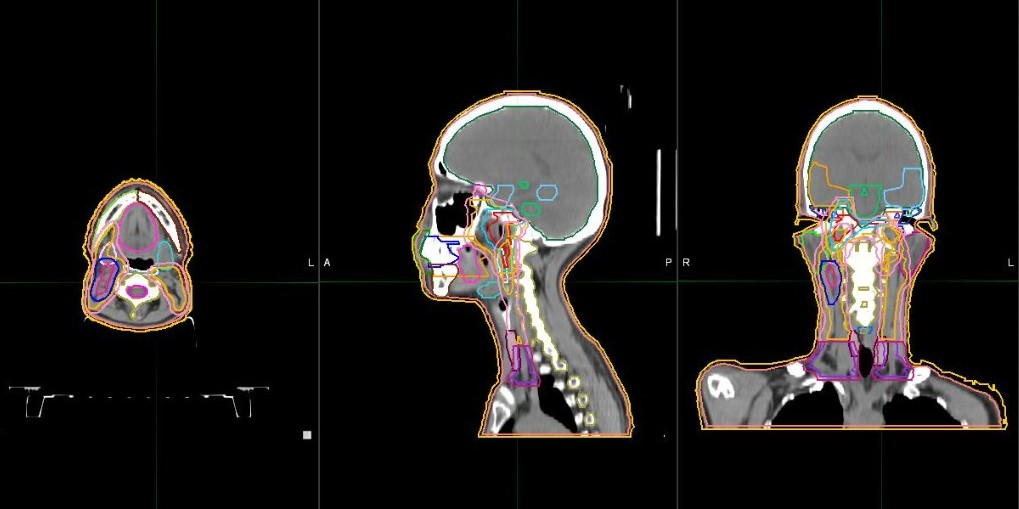

Supplement: Supplementary file 2 — Supplementary Fig. 1 CT scan images of one of the patients with delineated boundaries in the treatment planning system, including transverse, sagittal, and coronal views) [file 405_2023_8173_MOESM2_ESM.png]
